# Going around in a Circle: A Norwegian Study of Suicidal Experiences in Old Age

**DOI:** 10.1155/2014/734635

**Published:** 2014-12-09

**Authors:** Anne Lise Holm, Anne Lyberg, Ingela Berggren, Sture Åström, Elisabeth Severinsson

**Affiliations:** ^1^Centre for Women's, Family and Child Health, Faculty of Health Sciences, Buskerud and Vestfold University College, P.O. Box 235, Kongsberg, Norway; ^2^Department of Nursing, Health and Culture, University West, 461 86 Trollhättan, Sweden

## Abstract

Depression has repeatedly been found to be a risk factor for completed suicide, particularly when coupled with a pervasive sense of hopelessness. The aim of this study was to evaluate depressed older persons' suicidal experiences. Data were collected by means of individual in-depth interviews with nine informants living in two districts of Norway. A hermeneutic analysis was performed. One main theme: Going around in a circle and two themes: being alone without meaning in life and struggling to achieve reconciliation emerged from the analysis. An important implication for mental healthcare practice is the need to develop a person's ability to shape and take control of her/his life. The healthcare organisation must be committed to a plan that sets out strategies enabling suicidal individuals to avoid the negative experience of meaninglessness. It was concluded that suicidal depressed elderly persons need help to escape from their desperate situation. More research is urgently required in order to prevent suicide in depressed elderly persons whose emotional pain is unbearable.

## 1. Introduction

Suicide accounted for almost 32,000 deaths in the United Stated in 2004 [1]. The lethality of suicide attempts increases with age [2, 3]. The reason might be that older individuals who attempt suicide have a stronger wish to die than their younger counterparts [3]. According to Alexopoulos et al. [4], suicidal ideation and depression are two major risk factors for late-life suicide as well as targets for prevention. Late-life depression is common in primary care settings, affecting at least 5 to 10% of older persons [5], although it remains underdetected in those who live in their own home [6, 7]. In both earlier and recent studies, depression has been repeatedly found to be a risk factor for completed suicide, particularly when coupled with a pervasive sense of hopelessness [8, 9], which is more likely to lead to suicide than depression alone [10]. Research has revealed associations between late-life suicide ideation and intense psychological pain, including depression, poor social integration, and physical health problems [11–13]. Suicidal experiences of meaninglessness have been described as a state of psychache [14], human suffering [15], and emotional pain [16]. Many individuals are ambivalent about completing suicide, as the wish to live and the wish to die wage a battle [17]. There is an urge to escape from the pain of living and at the same time a desire to live [18].

**Figure 1:**
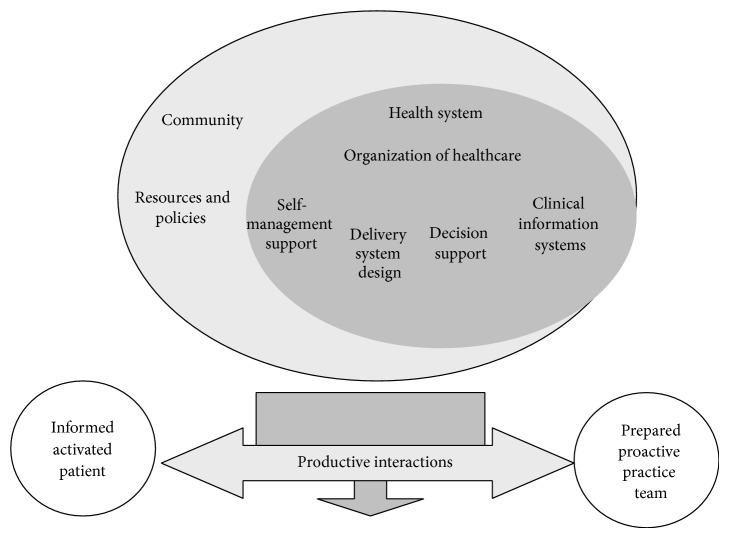
The Chronic Care Model (CCM) [24].

Stressful life events have been found to have a significant correlation with increased risk of suicide in older adults [19]. Such stressors include political and economic changes, bereavement, separation from family and friends, interpersonal problems, and shame. Lack of social support was associated with higher levels of depression and suicidal ideation in a study by Vanderhorst and McLaren [13], while Jahn and Cukrowicz [20] found that perceived burdensomeness may explain the link between depression and suicide ideation in older persons. Burdensomeness means a sense of thwarted belongingness or a lack of social connectedness through meaningful relationships. Heisel and Flett [12] indicated that suicide ideation was positively associated with depression and the number of self-reported physical health problems and negatively associated with the domains of well-being including positive relationships with others, self-acceptance, and perceived meaning in life. Lynch et al. [21] suggested that suicide prevention efforts in older persons might be improved by targeting emotional inhibition in affectively intense and reactive older individuals. Bruce et al. [22] tested the impact of a primary care-based collaborative suicide intervention on the reduction of risk factors for suicide in later life. Other primary care-based collaborative programmes for depression propose various strategies for reducing suicidal ideation [23] and improving the outcome of major depression in older persons [4]. The Chronic Care Model (CCM) (Figure 1) has been used in several studies as a conceptual model for quality improvement. It comprises six components: (i) community resources and policy; (ii) the health system and the organization of healthcare; (iii) self-management support; (iv) delivery system design; (v) decision support; and (vi) clinical information systems [24]. Ability to manage one's own life has been outlined in self-management literature and research [25, 26]. Thus depressed elderly persons need to be empowered to manage their suicidal experiences and take charge of their life [26]. It has been found that a collaborative team approach reduces the risk of suicide among depressed older persons in the community [4, 22–24].

Two studies have revealed that older people experienced the final phase of life as a burden and seemed to accept death [27, 28]. Rurup et al. [29] stated that the older persons in their study gradually developed suicidal ideation after a life full of adversity and as a consequence of aging, illness, or recurring depression. Bonnewyn et al. [30] described that life and the self were disrupted after bereavement, causing loneliness, loss of control, and unwillingness to continue living. Holm et al. [31] reported that depressed older people's narratives recounted their struggle with memories of loss, grief, abuse, and violence that they had experienced as children, adolescents, and/or adults. In addition, greater problems arise when there is a gap between healthcare systems, lack of a collaborative team model among healthcare professionals, and inadequate methods for facilitating self-management [31]. Suicide among elderly persons seems to be increasing worldwide [1–5]. The aim of this study was to evaluate depressed older persons' suicidal experiences.

## 2. Methods

### 2.1. Design

This follow-up study represents the final phase of a larger investigation aimed at implementing and evaluating the CCM for depressed older persons in the community [26, 32]. In 2013 our research team conducted exploratory studies on interview data to identify successful implementation with the aim of improving the care of suicidal and depressed elderly persons [26, 31, 32]. Implementation strategies as well as theoretical and methodological developments relevant to the CCM and mental health in the context of elderly persons were employed. This follow-up study had a hermeneutic qualitative approach and design [33] in order to interpret the meaning of the lived experiences of being suicidal. A key assumption in this approach is that meanings can only be understood and interpreted in the context in which they occur, that is, through the lived experiences of those involved [34].

### 2.2. Participants

The participants comprised two men and seven women selected from a sample of 29 older persons resident in two districts in Norway. The other 20 subjects were excluded because their narratives did not include descriptions of being suicidal. The inclusion criteria were persons diagnosed with a depressive or mood disorder, able to understand and speak the Norwegian language, resident in a community in Norway, referred to community healthcare during the previous six months, over 60 years of age, and willing to speak about their experiences. Eight of the participants informed the researchers that they were treated with medications for depression and bipolar disorder. The participants had been interviewed 12 months previously and were willing to narrate about their experiences. Their mean age was 65 years (see Table 2) for demographic details and suicidal state). As many as seven out of the nine participants still experienced being in a crisis one year after the intervention.

### 2.3. Data Collection

Data were collected in 2013 by means of individual interviews with nine participants living in the community, which took the form of a dialogue between the researchers (ALH and AL) and the participants. During the first interview in 2012, some of the participants were asked if they would be willing to be interviewed about their situation after 6–12 months. Mental health nurses in the communities also asked some of the participants from the first interview (*N* = 29) who they regarded as suitable for a second interview, if they would be willing to be interviewed again. The participants lived in communities on the west and east coast of Norway. The interviews were held in the participants' home or in the offices of the two researchers. The participants were encouraged to narrate about whether they had considered death and suicide or wished to die during the previous 12 months. If they answered yes they were asked about their suicidal ideation and how they managed their daily life. The interviews, which lasted between 60 and 120 minutes, were audiotaped and transcribed verbatim.

### 2.4. Data Analysis

The nine interviews that constitute the data for this study were analysed using a hermeneutic approach [34] and Gadamerian-inspired research analysis by Fleming et al. [35]. According to Fleming et al. [35], new meaning and understanding emerge by constantly reading the text and moving back and forth between the whole and the parts. A summary of each interview was written in order to identify its meaning. During the first phase several statements emerged related to suicidal experiences. In this phase, the first author read through the interviews and designed a table that was sent to the coauthors. In the second phase the first author underlined all statements pertaining to the participants' suicidal experiences. She then read the summaries for a second time and wrote code-words in the text to facilitate the analysis process. The intention was to interpret the content hidden in the statements by going beyond the experiences described in the text to capture the meaning of the participants' words. The third phase involved grouping the items into clusters related to a particular theme. The themes that emerged were considered representative of the underlying meanings of the statements in the summaries. In this phase the author returned to the whole text in an attempt to expand its meaning. She reflected on a higher level of abstraction on what the participants meant rather than on what they actually said. The main objective was to search for patterns in as well as the underlying meaning of the text [35]. The fourth and final phase concerned identification of main themes that were representative of the authors' common understanding of the text (Table 1). Data saturation was obtained by the authors comparing and agreeing on the different themes and subthemes. Several meetings were held before they reached consensus.

### 2.5. Ethical Considerations

The Ethical Guidelines for Nursing Research in the Nordic Countries, Northern Nurses Federation [36], were adhered to. Approval for the study was granted by The Regional Ethics Committee of Western Norway (number 2010/2242) and the study followed the guidelines of the Declaration of Helsinki [37].

The interviews were conducted in a sensitive manner so as not to increase the older persons' feeling of being overwhelmed by reflections on and descriptions of their suicidal experiences. The participants were provided with detailed written information and signed a consent form. They were assured that their name and identity would not be disclosed and that they had the right to withdraw at any time. All data were stored in a locked and fireproof filing cabinet.

## 3. Results

One main theme: Going around in a circle and two themes emerged from the analysis: being alone without meaning in life and struggling to achieve reconciliation.

### 3.1. Going around in a Circle

Seven of the nine participants revealed having had suicidal thoughts during the previous year. However, they reported that their life situation had been worse. One explained that this state was like going around in a circle where there was no way out. Their thoughts increasingly circled around their situation until they could think of nothing else. One woman described experiences from the past that kept her inside the circle. She constantly thought about the abuse that her two daughters had suffered and found it impossible to describe her sense of guilt. Nothing in the previous year had alleviated her emotional pain. Her catastrophic situation often escalated as there were many family conflicts and she did not dare to come out of the dark.

“I sent my daughters Christmas presents, but they never thanked me. There is no point going around hating each other. However, I could have phoned and asked them if we could forget the past and have more contact.” (Female number 5)

Two of the participants narrated about their suicidal thoughts as follows.

“It is like going around in a circle, lying there in the dark unable to sleep. I find no pleasure in my activities earlier in life such as being politically active. I have lost all motivation. I have nothing to offer so there is no point even trying.” (Male number 8)

“My life is a mess. All the time I struggle to close the holes in my life. I cannot let anything out because it would make it even more difficult to close the holes and if I fail I am terrified of falling into the darkness again.” (Male number 3)

#### 3.1.1. Being Alone without Meaning in Life

Being alone without meaning in life included the subthemes: striving to control emotional pain and loss of close relationships. The participants reported that they often lived in a state in which they thought about how to take their own life, especially if they were alone. Being alone was experienced as a lack of meaning in life. This involuntary loneliness was described as a catastrophic state that increased their suicide ideation.

*Striving to Control Emotional Pain*. Some of the participants explained the importance of mastering their emotional pain, adding that it was essential to have someone with whom they could discuss their fears.

“I can understand why people want to die. It is important that the pain and anxiety do not control me. It is a miracle that I am alive today. However, I really try to control myself, but it is not easy when you wake up in a state of terrible anxiety.” (Female number 9)

*Loss of Close Relationship.* Some of the participants explained that they had experienced many losses during the past year, while others had a loss several years ago, after which their life no longer had any meaning. They revealed they had to struggle on their own with no one to talk to.

“In the past year I lost two of my best friends so I feel quite alone most of the time.” (Female number 7)

“Although my daughter died several years ago I still think of her as if it happened yesterday.” (Female number 4)

After a hospital stay another women stated the following:

“I tried to commit suicide twice, once in 2011 and again in 2012. I felt so terrible that I took an overdose. I had decided to escape from this world. But I became sick and threw up. And I really did not want to die so I called the ambulance.” (Female number 6)

One of the men explained the following:

“After the divorce my life has no meaning. I feel worthless sitting alone and watching TV. This is no life.” (Male number 8)

#### 3.1.2. Struggling to Achieve Reconciliation

The participants revealed that they had to go through the emotional pain alone. It was impossible to obtain medication to alleviate it. Their life was a struggle to achieve reconciliation. This theme comprises four subthemes: feeling of inadequacy; the pain of not being forgiven; feeling bitter and humiliated; and taking responsibility for one's life which can alleviate emotional pain.

*Feeling of Inadequacy*. The participants revealed that a great deal had happened during the previous year. They narrated different reasons for their increased anxiety and emotional pain including fear of being inadequate and unable to live up to the expectations and demands of others. One woman explained the following:

“The roof of our house was damaged and I was responsible for having it repaired. I could no longer sleep as I thought about the house all the time. I was terrified that my mother would be dissatisfied with me, which made me completely exhausted.” (Female number 4)

*The Pain of Not Being Forgiven*. Some of the participants experienced emotional pain and did not comprehend why their family was unable to forgive them. One woman said that she cannot understand why her daughter did not forgive her despite the fact that she forgave her father for the sexual abuse.

“The pain is killing me and strikes deep in my soul. She told me that she does not need me anymore because she has a new family. It broke my heart. I remember thinking that I no longer need to be here for her.” (Female number 5)

*Feeling Bitter and Humiliated*. Some of the participants experienced bitterness and humiliation related to their past and blamed themselves for everything. In the case mentioned above, the participant's two daughters were sexually abused by their father. She stated the following.

“I am unable to forget or reconcile myself with my past. I still experience that everything was my fault. The memories return when I am feeling down. I accuse myself of not being a good mother and blame myself for everything, although I know that it was not my fault. My situation is still the same today.” (Female number 5)

The bitterness had different faces and was expressed in various ways.

“I admit that I am bitter. I have no life anymore. I sit here and the only thing I can look forward to is being carried out in a coffin.” (Male number 8)

One participant related that his daughter made him sign a contract to guarantee a loan. When she was unable to meet the repayments the bank asked him for the money. He explained to the community psychiatric nurse that his life was over and he no longer wanted to go on living.

“I thought about how to end my life day and night and was admitted to a psychiatric centre in 2012, but I did not get any help there for this problem. I explained about the betrayal to which I was subjected and that I feel abused and humiliated by my daughter. The social worker phoned my daughter, who said she did not understand the problem.” (Male number 3)

*Taking Responsibility for One's Life Can Alleviate Emotional Pain*. Some of the participants stated that they occasionally managed to alleviate emotional pain but found it difficult to put into words.

“I really cannot explain this apart from comparing it to giving birth. After the birth you forget the pain. It was the same with the emotional pain too. And when it disappeared I felt so relieved that I nearly forgot what it was like.” (Female number 5)

Another said that she talked to herself about what might be helpful. She did not want others to take over her life as she preferred to manage alone. As long as she was able to make her own decisions she felt free.

## 4. Discussion

The main theme interpreted in this study, going around in a circle, concerns the depressed elderly persons' whole situation. The participants in our study did not seem to experience that their life situation changed for the better or became more satisfactory. Emotional, mental, and psychological pain (psychache) appear to have the same meaning as unbearable pain in the mind, heart, or soul, which increases the risk of suicide [14, 16, 38]. Philosophers, poets, and lay persons have written extensively about emotional pain, while the clinical literature in the area of psychology and psychiatry has made almost no attempt to define and explain it [38, 39]. Despite being intimately connected to the body, pain is conceptually elusive and can be distinguished from suffering, as it is a sensation that is intertwined with mental and cultural experiences. Research has revealed associations between late-life suicide ideation and intense emotional pain, including depression, poor social integration, and physical health problems [11–13]. A person suffering from unbearable emotional pain should be enabled to integrate suicidal experiences and reconstruct the loss or trauma by strengthening her/his self-management ability as described in the CCM [24]. Healthcare professionals must be trained to encourage suicidal persons to “tell their story.” One way to achieve this aim is to establish a well-functioning team based on the CCM model, where the case manager is responsible for follow-up, contact with different caregivers, and the development of strategies to enhance suicide prevention and self-management by adherence to a care plan, as well as for providing support to overcome sources of distress.

*Being alone without meaning in life* was described as an involuntary catastrophic state. Emotional pain and suffering seem to be a visible manifestation of existential aloneness that alienates the older person from her/himself. Self-management ability can be lost due to estrangement [26]. Healthcare professionals can help the depressed elderly person to reflect on why being alone hurts so much and how they can obtain more meaning in life. Loneliness has been associated with depression. Someone who can help the suicidal elderly person to reach out of loneliness might increase her/his ability to connect with other people. Acceptance of being alone has been found to be a strength and a step out of feeling lonely, described by Pierce et al. [40] as a recovery process that seems to imply a sense of overcoming the suicidal state. However, the participants in this study seemed to be in a state where they experienced a sort of invisible blanket between themselvesand the world. This involuntary aloneness can be a part of thwarted belongingness and constitute a burden [20], where the only thing left to do was to lessen the burden on significant others. Persons who find themselves in such a state may require help from mental health professionals to prevent passivity and apathy. Mental health professionals need to move from a traditional authoritative role to form partnerships with their patients [24]. A prerequisite for forming partnerships is that depressed older individuals must assume responsibility for their life, which the literature often terms self-management [24]. However, it has been found that self-management can lead to an unreasonable shift of responsibility to the patient [41]. In the CCM the coordinator is termed case manager, a role assumed by a nurse or physician who is responsible for creating a dialogue with the patient. Such a dialogue can reveal hidden power aspects about which one needs to be aware in order to prevent violation of the vulnerable depressed elderly persons' dignity. The case manager should increase understanding of the fact that although depressed elderly persons often feel powerless, they are also moral agents with their own values [42].

*Loss of close relationships* was described by several participants. Does the struggle to come to terms with loss present any opportunity for growth? Grief can be an emotionally exhausting experience that may increase suicidal thoughts. The experience of loss, at least in most Western contexts, may reflect a sense of going through difficult times, followed by a less difficult period. The suicidal experiences of loss were supported by Bonnewyn et al. [30] where the participants described a significant loss that had a tremendous impact on their life such as the death of a spouse, conflict with a child, or physical illness. After the loss, life no longer appeared worth living. As mentioned by Lynch et al. [21], cognitive ability is often reduced in older adults. Thus it is necessary to address the cognitive and emotional aspects of depression by introducing tasks to help older adults regain self-control and interrupt suicidal thoughts, which may in turn improve decision-making. It should be borne in mind that eight of the participants were treated with medications for depression and bipolar disorder, which could have triggered their suicidal behaviour as suicidal ideation is a common side effect of certain medications.

Searching for meaning seems to be a daily struggle, which in the CCM (Figure 1) is related to the loss of self-management ability. Joiner [43] proposed a theory of suicide, which consists of three variables, the first being that the desire for suicide arises when a person perceives her/himself as a burden on significant others. The second is that there is a sense of thwarted belongingness and the third, a lack of social connectedness through meaningful relationships. Understanding the competence of the other can provide an opportunity to become connected but demands the ability to manage one's life, which appears to be lacking. Acknowledging one's responsibility for the other can be impossible when one does not understand how to manage one's own life and emotional pain. Nevertheless, assuming responsibility for other people can strengthen self-management and provide an opportunity to escape the “shadows from the past” [31].

*Struggling to achieve reconciliation* revealed that the older persons' past traumatic experiences were still present [31]. The shadows or trauma from the past remained alive in their daily life. Reconciliation has been defined as establishing, overcoming, enduring, and purifying [44]. It is also understood as a desire for wholeness and meaning in life, an opportunity to reconcile fragmented memories, which can increase emotional pain [16]. New meaning enables the human being to overcome trauma, which can be a strategy for enduring emotional pain. Lack of reconciliation can lead to difficulty of loving and forgiving oneself as well as to bitterness, hostility, anger, and fear [44]. It appeared to be a feeling of being excluded from the possibility of relationships with other people and powerlessness to change the situation. Inability to obtain forgiveness was described as an endless struggle. As depressed older persons lack self-management ability, they need healthcare professionals to help them to reflect on and change their view of themselves, as the sense of being good enough could free them from feelings of guilt and open their eyes to viewing their life story in a different light. The CCM (Figure 1) provides healthcare professionals with an opportunity to grasp the urgent needs of suicidal older persons. Forgiveness might not be possible because one's guilty conscience increases the shame, thus hindering meaning and reconciliation. However, a person who searches for meaning and reconciliation can become aware of an opening to transform her/his fragmented memories into a new wholeness. Forgiveness seems to be an important part of reconciliation with other people. Therefore increasing self-management can be a way for healthcare professionals to help the suicidal person to see a way out of the endless circle around previous conflicts that cast shadows in her/his life. As forgiveness appears to be related to the act of speaking, emotions play a central role [45]. Feelings of inadequacy can be characterised as a sort of vulnerability related to expectations and rejection that increase emotional pain. Eisenberger et al. [46] demonstrated that two areas of the brain are involved in distressing feelings of social exclusion and respond in opposite ways to the degree of experienced emotional pain. Healthcare professionals require more coordinated efforts as outlined in the CCM (Figure 1) in order to increase suicidal elderly persons' ability to manage life without being overwhelmed by the pain of not being forgiven. It is vital to develop a range of strategies to provide experiences of inclusion and connection for depressed elderly persons in the community. Feeling bitter and humiliated increased these older persons' emotional pain. Despite being unaware of their sense of bitterness and humiliation, the older persons can become controlled by it. To overcome bitterness and humiliation, one has to surrender the emotional pain and let it go, which can be a way of promoting better control of one's life. Healthcare professionals need to empower suicidal older persons, thus endowing them with a sense of purpose in the sacred circle of life and death [47]. The liberating power of surrendering bitterness and humiliation can enable depressed elderly persons to survive emotional pain.

Taking responsibility for one's life in order to alleviate emotional pain was challenging for the participants as it increased the risk of suicide. It seems as if alleviating and surviving emotional pain can be related to assuming responsibility for oneself as well as for other people. In order to alleviate emotional pain and anxiety, it is necessary to increase self-management by strengthening one's social responsibility. The findings indicated the need to provide increased support to help suicidal elderly persons take responsibility for their life. One strategy is to become reconciled with their own history by forming the fragments into a story in which they are able to find meaning. As suggested in the CCM (Figure 1), healthcare professionals can encourage and empower suicidal elderly individuals by strengthening their self-management. Being able to accept one's story can be the first step to assuming responsibility for one's life and has been described as a way to alleviate emotional pain [16]. Healthcare professionals must act on these research recommendations and help suicidal elderly persons to take responsibility, thus alleviating their emotional pain. Responsibility implies that the suicidal elderly person is capable of taking part in the planning of goals, based on the notion that people are moral agents with their own values [42]. Being active and informed is also a step in the right direction towards responsibility and shared decision-making, although it can take a long time because every human being constructs things differently. However, the CCM (Figure 1) indicates that healthcare professionals should consider themselves a companion who is able to empower the patient by means of productive interactions. This can be related to the need for a shared decision-making model that can increase the trust between the team and the suicidal elderly person [47]. Such changes can strengthen optimism, control, well-being, and the pathways that lead to reconciliation.

## 5. Implications for Practice

Healthcare professionals need a new understanding that includes respect for the expertise that a person brings to the management of her/his condition. The core of being capable of taking responsibility for one's life seems to have an existential dimension of being in the world as well as related to freedom and dignity. Interventions that highlight the depressed elderly person's empowerment, participation, and involvement have been described in the CCM (Figure 1) and the literature [23–25]. Problems arise because the working methods are inadequate for supporting self-management [25].

Healthcare professionals must reflect on how best to help suicidal elderly persons engage in self-management and take responsibility for their life. However, achieving this aim can take time. Although these older persons have a great deal of contact with mental health nurses in the community who have taken part in an implementation programme, the nurses do not work according to the team model suggested by Wagner et al. [24]. They have no care manager to coordinate their work and functional leadership based on the principles of the CCM is lacking. When working according to a team model such as the CCM, one must respect and enhance patients' autonomy as well as maintain a high level of judgment and skill to increase their self-management ability and safety. The team needs to reach agreement on planned goals. Such goals can guarantee that suicidal elderly persons receive education about how to manage their daily life. Different prevention strategies are necessary, for example, a person they can phone when they are afflicted by suicidal ideation or serious problems that influence their daily life. When such patients are in a suicidal state, healthcare professionals must decide when to assume responsibility for them. This is a balancing act because of the older persons' vulnerability associated with previous trauma and violation that have been experienced as intrusive and have had consequences for their sense of dignity. Such experiences can dominate shared decision-making. One of the most important objectives of mental healthcare is the development of a person's ability to shape and take control of her/his life. Dignity is a core concept in nursing care, as care without dignity negatively influences recovery [48]. The healthcare organisation in some communities in Norway does not seem to be committed to a crisis plan with the necessary standards, which can be disastrous for suicidal elderly persons.

### 5.1. Study Limitations

This study has limitations. The sample is small and includes more women than men. A larger sample with more men could have produced different results. According to Gadamer [34], the hermeneutic circle requires an awareness of one's preunderstanding. The present researchers have long experience of mental health, intensive and critical care nursing, in addition to which they have conducted a great deal of research. The participants' descriptions are their interpreted experiences, influenced by culture and time.

As the participants' narratives were in Norwegian, translating the quotations into English can be considered an act of interpretation. Due to having been interviewed about the same issue several months previously, they were more willing to share their experiences on the second interview occasion. The narratives allowed the researchers to understand from a first person perspective, which can be an advantage, but might also influence the interpretation in a negative way. As a researcher one feels honoured to obtain such information, while at the same time being aware that one cannot follow up on the individual concerned. Thus one must consider one's role as a researcher and not as a mental health nurse. It is important to protect the participant by not allowing her/him to reveal too much, which she/he might later regret.

Trustworthiness is an essential part of the research process in qualitative interviews. Credibility can be seen as the striving to achieve a shared understanding of the phenomenon studied [33, 49]. As there is no neutral position in a qualitative study, we explored the text in an objective way in accordance with the concept of confirmability. A shared understanding was based on agreement about the themes and subthemes that emerged from the analysis. Transferability concerns the possibility of repeating the investigation in a different context [33, 49]. Due to the small sample size, transferability in terms of generalising the results to other contexts is considered impossible. However, qualitative research methods can enable us to go beyond our research subjects to a deeper reflection about life itself.

## 6. Conclusion

This study is important as it revealed that suicidal depressed elderly individuals need help immediately to emerge from their desperate situation. There is an urgent need for more research that can prevent suicide in depressed elderly persons suffering from unbearable emotional pain. A cry for help to survive was evident in the stories narrated by our informants. Healthcare professionals and researchers must take action to address this problem before it is too late.

## Figures and Tables

**Table 1:** Suicidal experiences in old age.

Going around in a circle	
Being alone without meaning in life	Struggling to achieve reconciliation
Striving to control emotional pain Loss of close relationships	Feeling of inadequacy The pain of not being forgiven Feeling bitter and humiliated Taking responsibility for one's life can alleviate emotional pain

**Table 2:** Demographic characteristics and suicidal experiences.

Number	Age	Living alone/widow/widower/divorced	Sex	Disorder	Suicidal history
1	67	Widow, living alone	Female	Depression	Suicidal ideation throughout her adult life but improved in the previous year.

2	62	Widow, living alone	Female	Depression	Suicidal ideation after the loss of sister and husband.

3	64	Divorced, living alone	Male	Depression	Suicidal ideation activated by feeling violated.

4	65	Divorced, living alone	Female	Bipolar	Suicidal ideation after the loss of her daughter ten years ago.

5	72	Divorced, living alone	Female	Depression	Suicidal ideation caused by a feeling of guilt related to the sexual abuse of her daughters.

6	67	Divorced, living alone	Female	Bipolar	Suicidal ideation over the course of the previous 25 years.

**7**	65	Divorced, living alone	Female	Depression	Suicidal ideation over the course of the previous twenty years.

8	65	Divorced, living alone	Male	Depression	Suicidal ideation over the course of the previous twenty years.

9	60	Divorced, living alone	Female	Depression	Suicidal ideation for 30–40 years, but recovered six years ago. Not suicidal today.
